# Mechanisms of Apoptotic Cell Death by Stainless Steel Nanoparticle Through Reactive Oxygen Species and Caspase-3 Activities on Human Liver Cells

**DOI:** 10.3389/fmolb.2021.729590

**Published:** 2021-09-24

**Authors:** Bader Almutairi, Daoud Ali, Khadijah N. Yaseen, Norah S. Alothman, Nouf Alyami, Hanouf Almukhlafi, Saad Alakhtani, Saud Alarifi

**Affiliations:** Department of Zoology, College of Science, King Saud University, Riyadh, Saudi Arabia

**Keywords:** CHANG and HuH-7 cells, apoptosis, oxidative stress, gene expression, stainless steel nanoparticles

## Abstract

**Background:** Nanoparticles are widely used in pharmaceutical, agriculture, and food processing industries and in many other fields. However, the effect of stainless steel nanoparticles (SSNPs) remains unclear. So in this study, we evaluate the effect of SSNPs’ toxicity on human liver (CHANG and HuH-7) cell lines over 24 and 48 h.

**Methods:** We have analyzed the quality, shape, and size of SSNPs using x-ray diffraction (XRD), energy dispersive x-ray (EDX) scanning electron microscope (SEM), and transmission electron microscope (TEM). The cytotoxicity and cell growth were determined by using the MTT and wound healing tests. The oxidative stress parameters were determined by measuring ROS generation and antioxidant enzymes, such as glutathione (GSH) and superoxide dismutase (SOD), due to SSNP exposure on human liver cell lines over 24 and 48 h. The confirmation of the apoptotic effect of SSNPs on livers cells was determined by the Western blot analysis for the expression of apoptotic proteins, such as Bax, bcl2, and p53, and real-time PCR for the expression of apoptotic genes, such as Bax, bcl2, caspase-3, and p53.

**Results:** We have observed the dose- and time-dependent cytotoxicity and apoptosis of SSNPs on both cells. The results showed that SSNPs induced cell toxicity, inhibited cell growth, GSH, and increased generation of intracellular ROS and SOD levels at higher concentrations of exposure in both cells. SSNPs showed an apoptotic activity with upregulation of Bax, caspase-3, and p53 and downregulation of the bcl2 gene expression in CHANG and HuH-7 cell lines. Moreover, the immunoblotting assay confirmed the apoptotic activity of SSNPs in cells.

**Conclusion:** In conclusion, these findings demonstrated that SSNPs showed toxic effects on human liver cells *via* activating the caspase-3 activity and they induced more toxicity in HuH-7 cells than in CHANG cells.

## Introduction

Nanotechnology has become one of the most rapidly growing areas of science and technology in all parts of the world. Thus, nanomaterials are applied in a wide range of areas such as cosmetics, electronics, and biomedical sciences. The product of nanotechnology has a big market, and its value was 39.2 billion dollars in 2016 and it will be 124 billion dollars in 2024 (Research and Markets, 2018). Engineered nanomaterials have great importance in consumer products and our daily life. Their novel physicochemical, thermal, and electrical properties facilitate their application in clothing, medicine, and cosmetics, thereby increasing the probability of human and environmental contact with these materials (Nel et al., 2006). The exposure of nanomaterials to the liver of animals and humans can occur through intentional and accidental means. The intentional application of nanomaterials might occur as a consequence of the consumption of nanoparticles containing drugs (Wim and Paul, 2008). The accidental exposure of nanomaterials to the liver occurs directly during manufacturing and disposing of used nanomaterial products (Gupta and Xie, 2018). The application of NPs in treating wounds and damage of skin accelerates penetration (Teow et al., 2011). So silver nanoparticles are frequently used in skin care products (Miethling-graff et al., 2014). The mechanism of human exposure and risk assessment of engineered nanoparticles, oxidative stress that is associated with decreased viability, the inhibition of the mitochondrial activity, and the initiation of apoptosis and cell death (Foldbjerg et al., 2009). Thus, nanomaterials induced cytotoxicity in cells due to the accumulation of metal ions (Fröhlich, 2013). The liver is an important organ of the human body and has a major role in physiology and detoxification. These concerns include the cytotoxicity of the liver, toxicity during accumulation in the liver for a long time, and metabolism with the potential of toxicity when present in the liver (Tsuji et al., 2006). NP-mediated toxicity involves various mechanisms, in particular the production of excess reactive oxygen species (ROS). As is well known, the mitochondrial dysfunction is the major source of ROS overload (Schumacker et al., 2014). De Prins et al. (El-Sayed et al., 2005) reported that ROS generation and apoptotic and inflammatory responses are the underlying mechanism of toxicity of environmental pollutants. Oxidative stress has mainly resulted from the generation of reactive oxygen species (ROS). Excessive ROS causes the imbalance of the antioxidant system in the body, which may induce lipid peroxidation and induce different types of enzymatic activities (Zhuang et al., 2018). This is the first study that reported the adverse effects of SSNPs on human liver cells. This study aimed at determining the toxic effects of SSNPs on human liver cells.

## Materials and Methods

### Chemical and Reagents

Stainless steel nanoparticles (SSNPs, Cr-Ni-Mo-Fe-Mn alloy, and 99.99%, 50 nm, Stock #: 316L) were purchased from US Research Inc., Houston, TX, United States. MTT [3-(4, 5-dimethylthiazol-2-yl)-2, 5-diphenyltetrazolium bromide] and 2, 7-dichlorofluorescein diacetate (H2-DCFH-DA) were purchased from Sigma-Aldrich (St. Louis, Missouri, United States). Dulbecco’s modified Eagle’s medium (DMEM), fetal bovine serum (FBS), and antibiotics were purchased from Gibco, United States.

### Characterization of SSNPs

The SSNPs were characterized using energy dispersive x-ray (EDX) scanning electron microscope and scanning electron/transmission electron microscope (SEM/TEM) (JEOL Inc., Tokyo, Japan) operated at 200 kV and x-ray diffraction (XRD) (Rigaku, Tokyo, Japan) operated at 9 kW and coupled with Smart Lab Guidance software (Smart Lab Studio II package software).

### Cells and Culture Conditions and Treatment of Nanoparticles

CHANG and HuH-7 cells were procured from American Type Culture Collection (ATCC), USA. These cells were subcultured in DMEM with 10% FBS and 10,000 U/ml antibiotics at a 5% CO_2_ incubator at 37°C. The cells at 80% confluence were subcultured into 96-well plates, 6-well plates, and 25-cm^2^ flasks according to the designed experiments.

CHANG and HuH-7 cells were sub-cultured overnight before exposure to SSNPs. The stock solution of SSNPs was made with double-distilled water at the rate of 1-µg NPs/µl DDW and diluted according to the experimental dosage (0–300 μg/ml). Cells not exposed to NPs were considered as controls in each experiment.

### 3-(4, 5-Dimethylthiazol-2-yl)-2, 5-Diphenyltetrazolium Bromide Test

The mitochondrial activity was determined by the MTT test (Alarifi et al., 2015). The MTT solution (100 µL) was mixed to each well in a final concentration of 0.5 mg/ml and further left for incubation for an additional 3.5 h. The formed formazan crystal was dissolved in isopropanol, and the absorbance was measured at 570 nm using a BioTek Epoch plate reader (BioTek Instruments, Winooski, VT, United States) and Gen5 software (version 1.09).

### Wound Healing/Scratch Test

The experiments were performed *in vitro*, measuring the cell migration after scratch with and without SSNPs on two cell lines, CHANG and HuH-7. These cell lines were first seeded into a 12-well tissue culture plate at approximately 540,000 cells/well and incubated overnight at 37°C and 5% CO_2_ in an incubator. We use a serial dilution of the SSNPs at 10, 50, and 100 μg/ml and exposed the cells overnight, which will allow the cells to reach 80% confluence. Then, with a sterile 0.1-ml pipette tip, a scratch was made on the culture plate to create a cell-free area. Finally, using an inverted microscope LeicaMC-170 HD camera (Leica, Germany), the scratch images were captured at 0, 24, 36, and 48 h at 200 × magnification. The time-lapse images were analyzed using ImageJ software. Each experiment was performed in duplicate. The relative migration ratio is calculated as described by Luanpitpong et al. (2010).

### Intracellular Reactive Oxygen Species Generation

The generation of intracellular ROS in CHANG and HuH-7 cells after exposure to SSNPs (0, 10, 50, 100, and 300 μg/ml) for 24 and 48 h was measured according to the methods described by Alzahrani et al. (2019). Shortly, 3 × 10^4^ cells were cultured in a black bottom culture plate (96 well) and incubated at 37°C in a CO_2_ incubator for 24 h for attachment. After exposure, the culture plates were washed with chilled PBS and 10-µM DCFH-DA was added in each well at 37°C for 40 min. After incubation, the plate was washed and the fluorescence intensity of dichlorofluorescein (DCF) was measured at 485-nm excitation and 520-nm emissions using the microplate reader (Synergy-H1; BioTek). Data were represented as the percent of fluorescence intensity relative to the control wells.

Another set of experiments (1 × 10^3^ cells/well in a 6-well transparent plate) was performed for analyzing the intracellular fluorescence using a fluorescence microscope (Olympus CKX 41; Olympus: Center Valley, Pennsylvania, United States), with images taken at 40 × magnification.

### Oxidative Stress

After treatment with NPs for 24 and 48 h, the cells were collected by 0.25% trypsin and split by ultrasonic crushing. After that, the cell samples were used for the detection of oxidative stress parameters, such as lipid peroxidation (LPO) and reduced glutathione (GSH), according to the instruction of Cayman kits.

The protein content was measured using the method described by Bradford (1976) with bovine serum albumin as the standard.

### Western Blotting

For Western blotting, 20 µg of protein was applied to the lanes of 4–12% Bis–Tris gels (Life Technologies), then blotted onto Immobilon-P membranes (Millipore, Bedford, MA, United States), and incubated with the relevant primary antibody. Appropriate species-specific conjugated secondary antibodies were commercially obtained (GE Healthcare, Tokyo, Japan). Proteins were detected using the ECL Prime Kit or the ECL Kit (GE Healthcare) with an Image Quant LAS 4000 system (GE Healthcare). All protein expression levels were normalized to the levels of the β-actin protein expression in each band.

### RNA Extraction, cDNA Synthesis, and Real-Time PCR

The RNA was reverse-transcribed using RT-PCR kits (Applied Biosystems, Foster City, CA, United States) with an oligo d (T) 16 primer under standard conditions. Real-time PCR amplification was performed using a Light Cycler 480 (Roche, Basel, Switzerland) and 2 ml of purified cDNA product, 0.5 ml of sense primer (10 pmol/ml), 0.5 ml of antisense primer (10 pmol/ml), 1 ml of Light Cycler Fast Start DNA Master SYBR Green I (Roche), and 0.8 ml of MgCl2 (25 mmol/L). Commercial glyceraldehyde phosphate dehydrogenase (GAPDH) primer sets were used for PCR amplification under the conditions recommended by the manufacturer (Table 1). GAPDH served as an internal reference gene, and the relative change was calculated by relative quantification, applying the formula 22DDCt.

**TABLE 1:** List of primer sequences of apoptotic genes.

Gene	Primer F sequence (5′->3′)	Primer R sequence (5′->3′)	Product size	Mt
Bcl2	ATG​TGT​GTG​GAG​AGC​GTC​AA	GGG​CCG​TAC​AGT​TCC​ACA​AA	143 bp	58
Bax	TGA​AGC​GAC​TGA​TGT​CCC​TG	CAA​AGA​TGG​TCA​CGG​TCT​GC	82 bp	58
TP53	TGA​CAC​GCT​TCC​CTG​GAT​TG	GCT​CGA​CGC​TAG​GAT​CTG​AC	83 bp	58
Caspase-3	CGG​CGC​TCT​GGT​TTT​CGT​TA	TCC​AGA​GTC​CAT​TGA​TTC​GCT	122 bp	58
GAPDH	AAT​GGG​CAG​CCG​TTA​GGA​AA	AAA​AGC​ATC​ACC​CGG​AGG​AG	133 bp	58

Reaction products were separated on 2% agarose gels.

### Statistical Analysis

All statistical analyses were performed using SPSS 26.0 software (IBM). Data expressed are mean and standard error (SE). Differences were analyzed using a one-way ANOVA 176 test with the least significant difference test. Values of *p < 0.05 were considered statistically significant.

## Results

### Characterization of SSNPs

**FIGURE 1:**
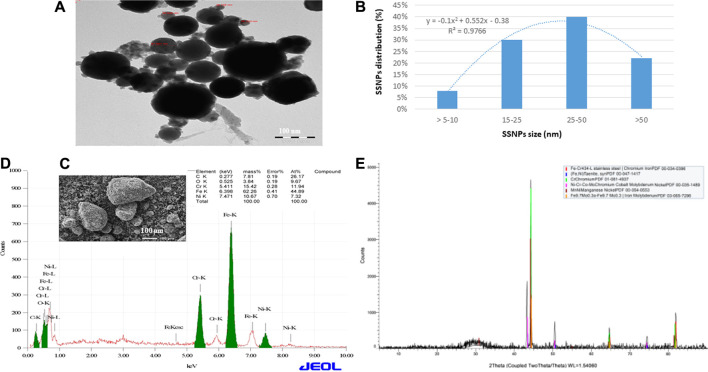
**(A)** TEM image of SSNPs. **(B)** Distribution of SSNPs in water. **(C)** SEM image of SSNPs. **(D)** Analysis of SSNPs by EDX. **(E)** XRD spectrum of SSNPs.

The size and shape of SSNPs were examined by using SEM and TEM, which showed that they were spherical and polygonal shaped with different sizes (Figures 1A–C). The average size of SSNPs was 43.2 ± 1.8 nm (Figures 1A,B). The quality of SSNPs was characterized by using EDX and XRD. We have observed Fe, Ni, Cr, and Co elements in EDX and XRD analysis (Figures 1C,E). Values of miller indices (h,k,l) for Fe, Ni, Cr, and Co of SSNPS in XRD planes are presented in Supplementary Figure S1. Figure 1E demonstrates the XRD pattern of the prepared SSNPs, which shows the crystalline nature of this material. The size of the crystal was determined from the XRD pattern using Scherrer’s equation (Patterson, 1939),

$$d=\frac{K\lambda }{\beta \cos\theta },$$

where D is the grain size, *λ* is the wavelength of the x-ray (1.54056 Å), β is the full width at half maxima of the diffraction peak (in radian).

The average element size corresponding to the maximum peak observed in XRD was found to be 50 nm. The existence of sharp structural peaks in XRD forms and the mean element size less than 50 nm suggested the nano-crystalline nature of the particles.

### Cytotoxicity

**FIGURE 2:**
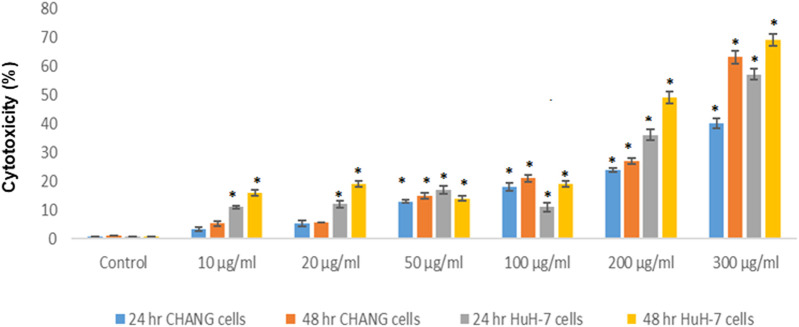
Cytotoxicity of SSNPs in CHANG and HuH-7 cells for 24 and 48 h, as evaluated by MTT assays. Each value represents the mean ± SE of three experiments. *n* = 3, *p < 0.05 vs. control.

The toxicity of SSNPs was measured by the MTT assay and SSNP-induced cell death in both cell lines. The result of the cytotoxicity is presented in Figure 2. SSNPs induced cytotoxicity in CHANG and HuH-7 cells in a time- and dose-dependent manner, but more toxicity was observed in HuH-7 cells than in CHANG cells (Figure 2). The toxicity of SSNPs at concentrations of 10, 20, 50,100,200, and 300 μg/ml was found to be 6, 9, 13,18, 24, and 40% for 24 h and 7.91, 9.4, 15,21, 27, and 63% for 48 h, respectively, in CHANG cells (Figure 2). The toxicity of SSNPs at concentrations of 10, 20, 50,100,200, and 300 μg/ml was found to be 10.95, 12, 16,11, 37, and 56% for 24 h and 15.6, 19, 14, 19, 49, and 69% for 48 h, respectively, in HuH-7 cells (Figure 2).

### Scratch Assay Results

**FIGURE 3:**
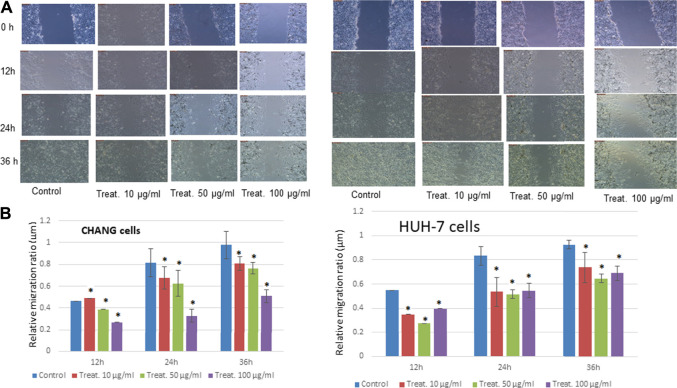
Treatment with steel nanoparticles decreases motility and invasion of CHANG and HuH-7 cells. **(A)** The wound healing/scratch test indicated that the motility of CHANG and HuH-7 cells was diminished by 12, 24 and 36 h of incubation with steel nanoparticles. **(B)** The percent wounded area filled in the wound healing/scratch test is shown as a graph. Data are shown as mean ± SE of six separate experiments. *p < 0.05 vs. control.

The physical injury was simulated in a monolayer of CHANG and HuH-7 cells, and the migration of cells was visually monitored until 36 h after the initial scratch (Figure 3). At 0 h, in both the control and dosed cell sets, scratches were visible with clear boundaries and with little to no cellular debris remaining in the scratch site after PBS rinse. As the time increased, the cell migration was evident in both sets as the width of the scratches became smaller and the boundary of the sites became blurred (Figures 3A,B). After 36 h, a mean closure of 90% was observed in the control set, while the dosed cells recorded a mean closure of 31% (Figures 3A,B).

### Oxidative Stress

**FIGURE 4:**
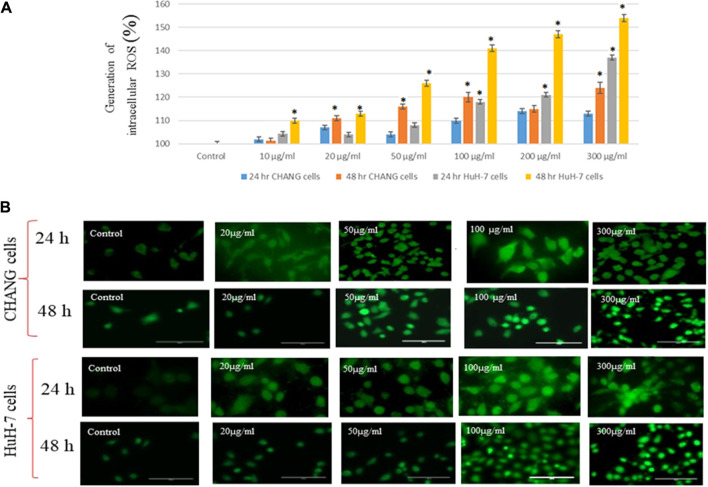
**(A)** Generation of intracellular ROS in CHANG and HuH-7 cells for 24 and 48 h due to SSNPs, as evaluated by the DCFDA fluorescence stain assay. **(B)** DCF-fluorescence intensity as a marker of ROS generation in CHANG and HuH-7 cells for 48-h exposure to SSNPs. Each value represents the mean ± SE of three experiments. *n* = 3, *p < 0.05 vs. control.

We observed the production of ROS in both cell lines after exposure to SSNPs. SSNPs induced too much generation of ROS in cells (2 × 10^5^/well) (Figure 4A). The intensity of the green fluorescence was observed to be maximum in HuH-7 cells than in CHANG cells at 300 μg/ml SSNP exposure (Figure 4A). The intensity of ROS production in both cells was measured using a fluorescence microplate reader. The generation of ROS in both cells after exposure to SSNPs was observed in a time- and dose-dependent manner (Figure 4B). The high intensity of the green fluorescence demonstrates more generation of ROS in cells (Figure 4B).

**FIGURE 5:**
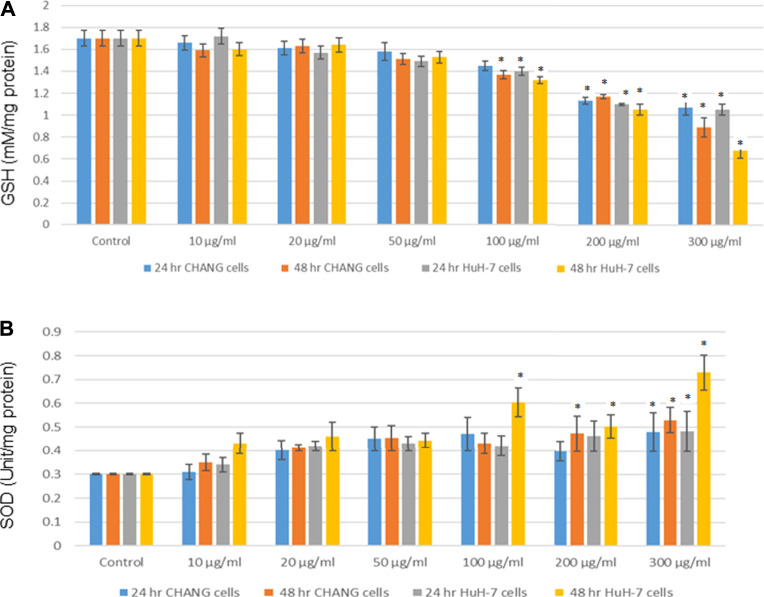
After exposure of SSNPs on CHANG and Huh-7 cells for 24 and 48 h. **(A)** Levels of GSH. **(B)** SOD in cells. Each value represents the mean ± SE of three experiments.*p < 0.05 vs. control.

GSH and SOD were determined and statistically analyzed with the untreated group. The SOD level was slightly increased in both cells (Figure 5B), and the GSH level was decreased after the NP exposure and much reduction was observed in HuH-7 cells (Figure 5A).

### Western Blotting of Apoptotic Proteins

**FIGURE 6:**
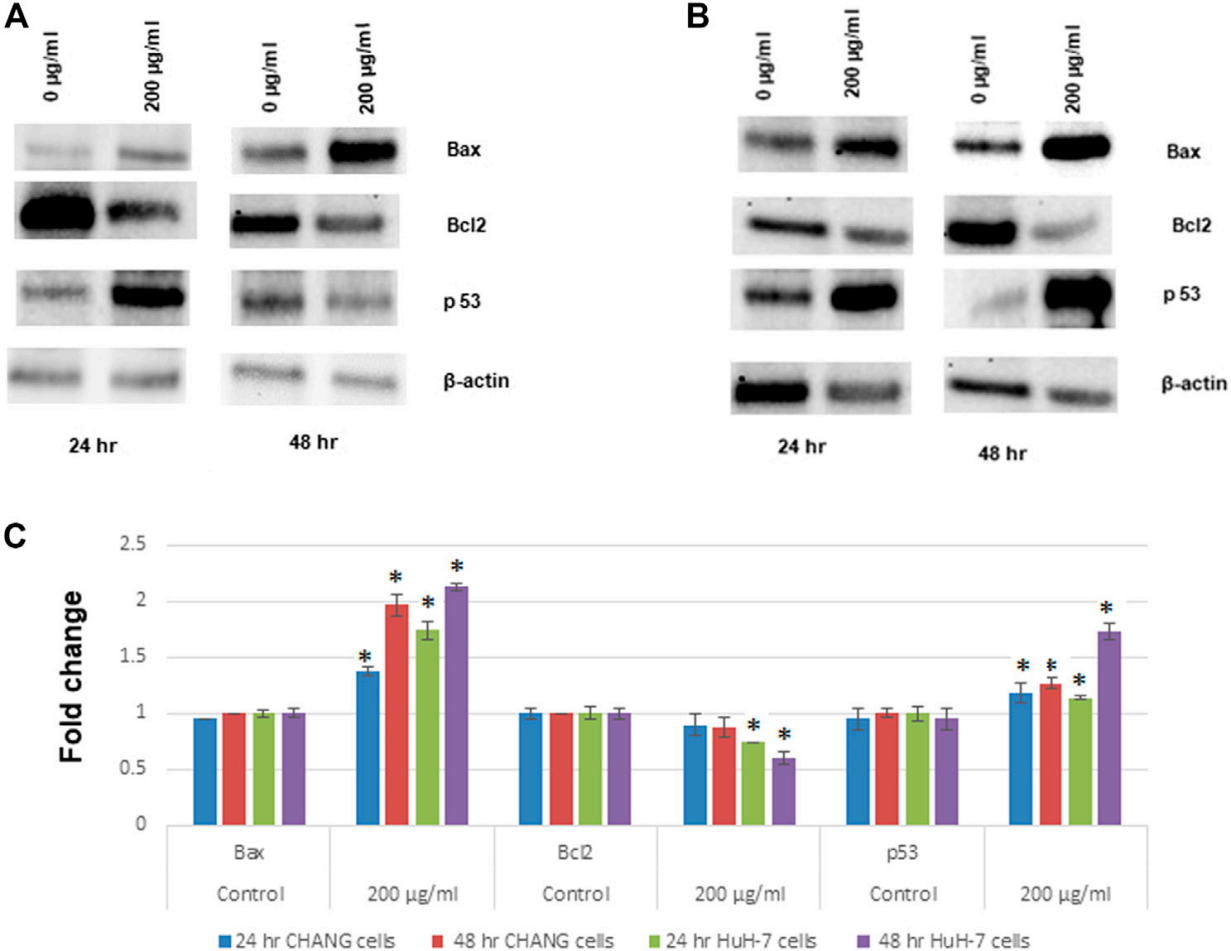
Western blot analysis of proteins involved in apoptosis: Bax, bcl2, and p53 expression levels. **(A)** CHANG cells. **(B)** HuH-7 cells. **(C)** Relative quantification of protein expression levels. β-actin was used as an internal control to normalize the data. Data represent mean ± SE of three experiments. *p < 0.05 vs. control.

The expression of apoptotic proteins in CHANG and HuH-7 cells due to exposure to SSNPs was observed using Western blotting. In both cells exposed to SSNPs, there is time- and dose-dependent upregulation of Bax and p53 levels (Figures 6A–C). In addition, the SSNP exposure decreases the expression level of bcl2 in a dose- and time-dependent manner (Figures 6A–C). In accordance with the Western blotting results, SSNPs exhibited the apoptotic effect in both cell lines.

### Expression of Apoptotic Gene Expression in Human Liver Cells

**FIGURE 7:**
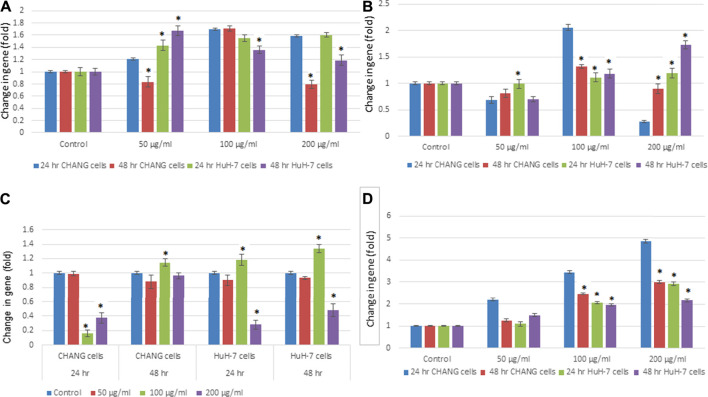
Expression of apoptotic genes in CHANG and HuH‐7 cells exposed to SSNPs for 24 and 48 h. Expression of **(A)** Bax, **(B)** bcl2, **(C)** p53, and **(D)** caspase‐3 gene in CHANG and HuH-7 cells for 24 and 48 h. Results are expressed in average ± SE of triplicate experiments. *p < 0.05 vs. control.

To examine the expression level of the apoptotic genes in human liver cell lines, RT-PCR analysis was performed. The maximum effect was seen after 48 h of exposure; SSNPs (200 μg/ml) showed downregulation of p53 (Figure 7C). The p53 gene was down-regulated in both cells, and much reduction was observed in HuH-7 cells than in CHANG cells (Figure 7C). Other apoptotic genes, such as Bax, bcl2, and caspase-3, were expressed in CHANG and HuH-7 cells (Figures 7A,B,D).

## Discussion

The application of alloy nanoparticles has different properties, such as optical, magnetic, and electrical properties, compared to single metal nanoparticles; it is used to improve NPs’ biocompatibility and increase their stability. However, the alloy nanoparticles, e.g., SSNPs, induce toxicity due to the selective interaction with various types of cells and biological molecules (Jeevanandam et al., 2018). At present, NPs are important materials for humans and animals. NPs have beneficial as well as harmful effects on the health of human and animal. In this experiment, we have analyzed the toxicity of SSNPs on human liver cells (CHANG and HuH-7 cells). The size and quality of SSNPs were examined using SEM, TEM, EDX, and XRD before the experiment because the size and surface area of NPs play an important role, generally determining the distinctive mechanism of the SSNP interaction with target cells (Auría-Soro et al., 2019). The microparticles are bigger than nanoparticles; as a consequence, nanoparticles enter cells and induced more toxicity than microparticles that cannot enter cells (Huo et al., 2014).

The cytotoxicity of NPs may delay due to the cell division, protein synthesis, and activation of pro-inflammatory genes. Chen et al. (2010) reported that the osteogenic differentiation of stem cells activates the synthesis of signal molecules and tumor antigens were suppressed due to iron oxide NPs. In addition, the interaction of NPs with the cell induces the expression of the respective genes to inhibit protein synthesis (Wang et al., 2017). In this study, we have found that the toxicity is low with a lower concentration of SSNP exposure. This study also showed that cell viability is more inhibited in HuH-7 cells than in CHANG cells after exposure to SSNPs. Oxygen (O_2_) is needed to generate energy in mitochondria (Halliwell and Gutteridge, 2006). The O_2_ gas is toxic and mutagenic to mitigates its effects, aerobes have evolved antioxidant defenses system (Halliwell and Gutteridge, 2006). In this experiment, we have investigated the generation of intracellular ROS and showed that its level increases in a dose- and time-dependent manner and, subsequently, that the production of ROS was more in HuH-7 cells than in CHANG cells.

NPs induced free radicals, which may damage the cells through the oxidative stress mechanism. The production of ROS damages the plasma membrane of cells by the development of the MDA compound due to the lipid peroxidation process (Ayala et al., 2014). The level of LPO was higher in HuH-7 cells than in CHANG cells. ROS has a major role in various cellular mechanisms, such as cell cycle, cell proliferation, and gene expression, and ultimately the mechanism of cell growth was stopped or cell death occurred (Almutairi et al., 2020). We have observed that HuH-7 cells are more susceptible to SSNPs than CHANG cells. NPs possess special characters (size, surface area, shape, solubility, and aggregation status) that associate with their capability to produce ROS (Nel et al., 2006; Ray et al., 2009). Microparticles and fibers induced inflammation in lung cells (Donaldson and Tran, 2002). The apoptotic potential of SSNPs in both cells was analyzed by FACS analysis. The maximum apoptotic cells were gated in HuH-7 cells than in CHANG cells at higher concentrations. The stimulation of caspase-3 was accompanied by the downregulation of bcl2 and the upregulation of Bax proteins. All these events indicated the signs of apoptosis, which was observed more in A549 cells after exposure to NPs.

## Conclusion

In conclusion, excessive intracellular ROS was produced due to SSNP exposure. Oxidative stress plays a major role in SSNP toxicity in human liver cells. ROS induces liver cell death *via* apoptotic process pathways and is associated with the toxicity of SSNPs in liver cells. In the future, we will do studies on the mechanism of toxicity due to SSNPs in *in vivo* models.

## Data Availability

The original contributions presented in the study are included in the article/Supplementary Material; further inquiries can be directed to the corresponding author.
