# Supplementary material for: Mechanisms of Apoptotic Cell Death by Stainless Steel Nanoparticle Through Reactive Oxygen Species and Caspase-3 Activities on Human Liver Cells
Source: Front Mol Biosci. 2021 Sep 24;8:729590. doi: 10.3389/fmolb.2021.729590 (PMC8497807; doi:10.3389/fmolb.2021.729590)
Supplement: Supplementary file 1 [file DataSheet1.PDF]

XRD planes of SSNPs

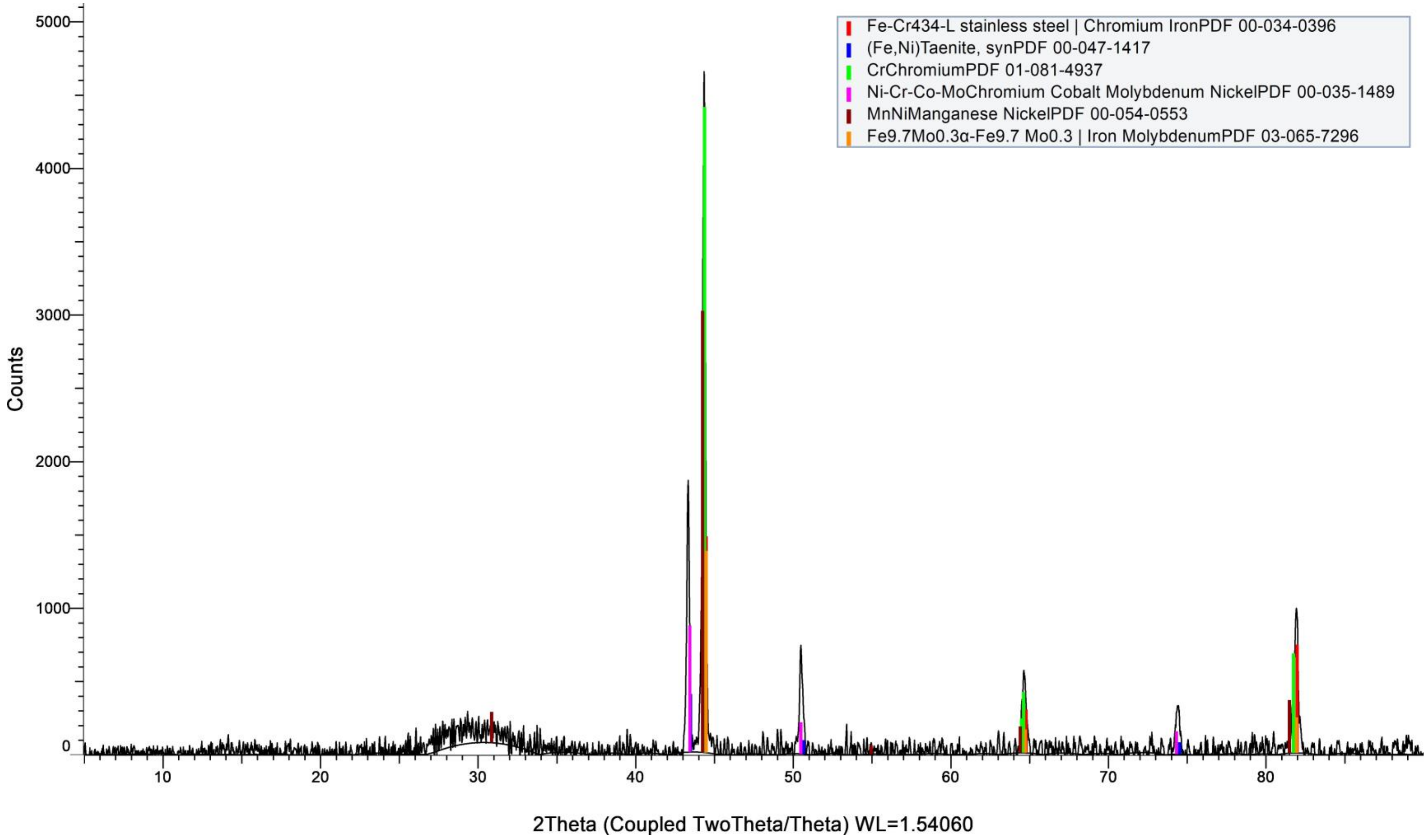

Pattern: PDF 00-034-0396 Radiation: 1.54060 Quality: Indexed

**Formula**	Fe-Cr
**Name**	Chromium Iron
**Name (mineral)**	
**Name (common)**	434-L stainless steel
**Status**	Primary
**Ambient**	Yes

d	2θ	I fix	h	k	l
2.03500	44.485	101	1	1	0
1.43800	64.779	21	2	0	0
1.17430	81.986	51	2	1	1
1.01700	98.475	19	2	2	0
0.90950	115.763	31	3	1	0
0.83020	136.204	13	2	2	2

**Lattice:**	Cubic
**S.G.:**	Im-3m (229)
**Mol. weight =**	110.69
**Volume [CD] =**	23.79
**Dx =**	
**Dm =**	
**I/Icor =**	0.590
**a =**	2.87600
**a/b =**	1.00000
**c/b =**	1.00000
**Z =**	1

Analysis: Spectroscopic analysis (wt %): Fe 80.10, Cr 11.70, Si 0.59, Mn 0.09, Ag 0.01, Mo 1.39, Ni 0.33, P 0.01, plus trace elements
Warning: Lower quality mark was set by the editor

Primary Reference
Publication: ICDD Grant-in-Aid
Authors: Pfoertsch et al., Penn State Univ., University Park, PA, USA.

**Radiation:**	CuKα
**Wavelength:**	1.54060
**SS/FOM:**	F(6)= 69.6(0.0144, 6)
**Filter:**	M
**d-spacing:**	

Pattern: PDF 00-047-1417 Radiation: 1.54060 Quality: Blank

**Formula**	(Fe,Ni)
**Name**	Iron Nickel
**Name (mineral)**	Taenite, syn
**Name (common)**	awaruite
**Status**	Primary
**Ambient**	Yes

d	2θ	I fix	h	k	l
2.07900	43.495	101	1	1	1
1.80000	50.674	25	2	0	0
1.27200	74.541	21	2	2	0
1.08500	90.462	12	3	1	1
1.03800	95.821	8	2	2	2
0.90000	117.716	3	4	0	0
0.82600	137.677	9	3	3	1
0.80500	146.232	6	4	2	0

**Lattice:**	Cubic
**S.G.:**	Fm-3m (225)
**Mol. weight =**	55.85
**Volume [CD] =**	46.56
**Dx =**	
**Dm =**	
**I/Icor =**	-1.000
**a =**	3.59750
**a/b =**	1.00000
**c/b =**	1.00000
**Z =**	4

Additional Patterns: To replace 00-023-0297
ANX: N
General Comments: Cell parameter generated by least squares refinement
Sample Preparation: Prepared by solid-state reaction at 800 C for 100 hours
Unit Cell: Reference reports: a=3.5949(5)
Warning: Lines with abs(delta 2Theta)>0.2 DEG
Unit Cell Data Source: Powder Diffraction

Primary Reference
Publication: Metall. Trans. A
Detail: volume 23, page 3177 (1992)
Authors: Morra, M., Ballinger, R., Hwang, I.

**Radiation:**	CuKα
**Wavelength:**	1.54060
**SS/FOM:**	F(8)= 9.6(0.1042, 8)
**Filter:**	M
**d-spacing:**	

Pattern: PDF 01-081-4937 Radiation: 1.54060 Quality: Prototyping

**Formula**	Cr
**Name**	Chromium
**Name (mineral)**	
**Name (common)**	
**Status**	Primary
**Ambient**	Yes

**Lattice:**	Tetragonal	**Mol. weight =**	52
**S.G.:**	I4/mmm (139)	**Volume [CD] =**	23.98
		**Dx =**	7.2
		**Dm =**	
		**I/Icor =**	6.980

**a =**	2.88200	**Z =**	2
**c =**	2.88700		
**a/b =**	1.00000		
**c/b =**	1.00173		

ANX: N
Analysis: Cr1
Formula from original source: Cr
ICSD Collection Code: 625712
Calculated Pattern Original Remarks: Becomes cubic above Neel-point (393 K)
Temperature of Data Collection: 300 K
Minor Warning: No R factors reported/abstracted
Wyckoff Sequence: a (I4/MMM)
Unit Cell Data Source: Single Crystal

Primary Reference
Publication: Calculated from ICSD using POWD-12++
Publication: Dokl. Akad. Nauk SSSR
Detail: volume 237, page 79 (1977)
Authors: Baklanova, L.M., Larkov, L.N., Ustinov, I., Chuistov, K.U.

**Radiation:**	CuKα1	**Filter:**	Not specified
**Wavelength:**	1.54060	**d-spacing:**	
**SS/FOM:**	F(9)= 56.8(0.0132, 12)		

d	2θ	I fix	h	k	l
2.03965	44.378	1000	1	0	1
2.03965	44.378	1000	1	1	0
1.44350	64.503	54	0	0	2
1.44100	64.628	95	2	0	0
1.17691	81.765	155	1	1	2
1.17691	81.765	155	2	1	1
1.01982	98.108	41	2	0	2
1.01894	98.222	25	2	2	0
0.91279	115.108	29	1	0	3
0.91153	115.358	51	3	1	0
0.91153	115.358	51	3	0	1
0.83244	135.442	22	2	2	2

Pattern: PDF 00-035-1489 Radiation: 1.54060 Quality: Indexed

Formula Ni-Cr-Co-Mo
Name Chromium Cobalt Molybdenum Nickel
Name (mineral)
Name (common)
Status Primary
Ambient Yes

d	2θ	I fix	h	k	l
2.08100	43.451	100	1	1	1
1.80590	50.497	25	2	0	0
1.27500	74.336	19	2	2	0
1.08800	90.144	14	3	1	1
1.04210	95.323	7	2	2	2

Lattice: Cubic
S.G.: Fm-3m (225)

Mol. weight = 230.67
Volume [CD] = 46.98
Dx =
Dm =
I/Icor = -1.000

a = 3.60820
a/b = 1.00000
c/b = 1.00000
Z = 1

Analysis: Nominal chemical analysis: Ni 53.42, Cr 18.65, Co 10.78, Mo 9.72, Ti 3.21, Fe 2.59, Al 1.43, C 0.086, Si 0.060, Mn 0.050, B 0.007, S 0.002
General Comments: Known as Rene 41
Sample Source or Locality: Specimen provided by Tempres, Division of Leco
Warning: Lines with abs(delta 2Theta)>0.06 DEG

Primary Reference
Publication: ICDD Grant-in-Aid
Authors: Pfoertsch, Ruud, Penn State University, University Park, Pennsylvania, USA.

Radiation: CuKα
Wavelength: 1.54060
SS/FOM: F(5)= 22.8(0.0438, 5)

Filter: M
d-spacing:

Pattern: PDF 00-054-0553 Radiation: 1.54060 Quality: Indexed

**Formula**	MnNi
**Name**	Manganese Nickel
**Name (mineral)**	
**Name (common)**	
**Status**	Primary
**Ambient**	Yes

d	2θ	I fix	h	k	l
2.89355	30.878	7	1	0	0
2.04481	44.260	100	1	1	0
1.66979	54.944	3	1	1	1
1.44523	64.416	7	2	0	0
1.18001	81.505	13	2	1	1
1.02278	97.727	4	2	2	0
0.91438	114.795	3	3	1	0
0.83496	134.607	2	2	2	2

**Lattice:**	Cubic
**S.G.:**	Pm-3m (221)
**Mol. weight =**	113.63
**Volume [CD] =**	24.18
**Dx =**	
**Dm =**	7.804
**I/Icor =**	-1.000
**a =**	2.89170
**a/b =**	1.00000
**c/b =**	1.00000
**Z =**	1

General Comments: Metastable phase. An equilibrium exists above 938 K
Processing Information: Rietveld refinement
Sample Preparation: Arc-melted. Powder prepared by machine milling
Temperature of Data Collection: 297 K
Warning: Lines with abs(delta 2Theta)>0.06 DEG

Primary Reference
Publication: ICDD Grant-in-Aid
Authors: Kimmel, G., Vemuganti, S., Kattumenu, R., Materials Science and Engineering, Western Michigan Univ., Kalamazoo, MI, USA.

**Radiation:**	CuKα1
**Wavelength:**	1.54060
**SS/FOM:**	F(8)= 24.9(0.0292, 11)
**Filter:**	F
**d-spacing:**	

Pattern: PDF 03-065-7296 Radiation: 1.54060 Quality: Blank

**Formula**	Fe9.7Mo0.3
**Name**	Iron Molybdenum
**Name (mineral)**	
**Name (common)**	α-Fe9.7 Mo0.3
**Status**	Primary
**Ambient**	Yes

d	2θ	I fix	h	k	l
2.03470	44.492	999	1	1	0
1.43875	64.741	117	2	0	0
1.17473	81.949	177	2	1	1
1.01735	98.429	47	2	2	0
0.90994	115.673	64	3	1	0
0.83066	136.045	18	2	2	2

Lattice: Cubic
S.G.: Im-3m (229)

a = 2.87750
a/b = 1.00000
c/b = 1.00000
Z = 0

Mol. weight = 570.48
Volume [CD] = 23.83
Dx = 7.95
Dm =
I/Icor = 11.040

NIST M&A collection code: L 28437 13541 0
Sample Preparation: Alloys were nonconsumably arc-melted, The ingots were then heated to 1073K under argon, forged to 3/4-in. rounds, cleaned by sand blasting, and swaged to 1/2-in. rounds
Calculated Pattern Original Remarks: sample composition is Fe32.3Mo; unit cell dimension taken from figure
Temperature Factor: TF was not given, B set to 1.000 for calc
Temperature of Data Collection: 21 C
Minor Warning: No e.s.d reported/abstracted on the cell dimension. No Rfactor reported/abstracted
Significant Warning: unit cell dimension taken from figure
Unit Cell Data Source: Powder Diffraction

Primary Reference
Publication: Calculated from NIST using POWD-12++
Publication: Trans. Met. Soc. AIME
Detail: volume 236, page 76 (1966)
Authors: Abrahamson, E. P., Lopata, S. L.

Radiation: CuKα1
Wavelength: 1.54060
SS/FOM: F(6)= 999.9 (0.0001, 6)

Filter: Not specified
d-spacing:
